# Colonial Hospital Laws

**Published:** 1920-07-24

**Authors:** 


### LEGAL NOTES FOR INSTITUTIONAL WORKERS.

### Colonial Hospital Laws.

The instructive and valuable Review of recently enacted Legislation of the British Empire and certain foreign countries, lately issued by the Society of Comparative Legislation, records some interesting enactments affecting hospital administration.

One of the Statutes of North Alberta (Canada) provides that the organised portions of the province—that is, the areas incorporated as local improvement districts, rural municipalities, villages, towns, and cities (of a population of less than 500) will be grouped into hospital districts, and these organisations may unite to provide hospital accommodation within such hospital district for the people living within its boundaries. This provision may be made by an arrangement with an existing hospital, or by the erection or purchase of a hospital to be operated under the directions of the hospital district. The business of the hospital district will be administered by a board appointed by the Councils of the organisations within the district. The regulations which will govern the operation of these hospitals, as well as the charges made for such accommodation, are matters which will be dealt with by the Hospital Board. A hospital district may make arrangements whereby all residents will be given free hospital accommodation and medical attendance at the expense of the municipalities, such expense to be met out of the revenue derived from the taxes levied; or the arrangements may simply provide for hospital accommodation, to be paid for by the municipality, leaving the doctor's fees to be paid by the individual. The rate of taxation which may be levied for capital expenditure in connection with any hospital scheme is limited to a maximum of two mills on the dollar. From information available as to the expense attached to the operation of a hospital of this kind this amount will, no doubt, provide ample funds.

This legislation has been placed on the Statute Book after very careful consideration, and reference to the different organisations interested—namely, the Rural Municipality and Local Improvement District Association, the United Farm Women of Alberta, the United Farmers of Alberta, and the Free Hospitals League—for their consideration before it became law. It should be of great benefit, more particularly to the rural communities, by placing in their hands the power whereby the electors may make provision for obtaining for the people living in the country medical attention without undue expense, and without requiring them to make long journeys.

The Transvaal Government has passed legislation restricting the working hours of nurses in provincial hospitals to not more than six days, or six nights, in any one week; limits night-duty to eleven hours; and prevents nurses being on night-duty for more than three calendar months in succession, subject, however, to excess duty in cases of urgency, provided cases of excess time and reasons therefor are certified by the Superintendent or Matron, and returns thereof forwarded monthly to the Administrator.

The Hospital Committees' Ordinance (Transvaal) of 1915, which provided for the election of Hospital Committees by local authorities, has been repealed, and the old system of nomination by the Provincial Administration restored, the new Provincial Government (elected in 1917) returning to the view that, as the province finds the bulk of hospital revenues, it should retain complete control. At the same time power is taken to establish a pension fund for hospital employees, and to compel contribution thereto.

One of the Ordinances of Southern Nigeria makes hospital fees payable in respect of a shipmaster or seaman recoverable from the master, owner, consignee, or charterer, or their agent. The payer may retain the amount from money received on account of the ship. Section 5 imposes an obligation on employers of labour to pay, in certain cases, the charges for services rendered and medicines supplies to their servants in Government hospitals.

We think our readers may be interested in having their attention called to these experiments in legislation. They are certainly worthy of studious consideration. Whether any of them can be adopted here is a matter for our legislators and those on whom they depend for advice and suggestions.

